# Impact of Dronedarone on Early Recurrence After Catheter Ablation in Patients With Nonparoxysmal Atrial Fibrillation

**DOI:** 10.1155/cdr/2585953

**Published:** 2025-11-14

**Authors:** Xianglin Long, Han Lv, Jingliang Zhang, Xiaoyan Wang, Shan Tu, Xu Deng, Lixiong Zeng, Wenzhi Luo, Fei Ye, Zhihui Zhang

**Affiliations:** Department of Cardiology, The Third Xiangya Hospital of Central South University, Changsha, China

**Keywords:** dronedarone, early recurrence, nonparoxysmal atrial fibrillation, radiofrequency ablation

## Abstract

**Objective:**

This study is aimed at comparing the efficacy and safety of dronedarone, amiodarone, and propafenone in preventing early recurrence of atrial arrhythmias during the blanking period following catheter ablation in patients with nonparoxysmal atrial fibrillation.

**Methods:**

We retrospectively analyzed 408 patients with nonparoxysmal atrial fibrillation who underwent their first catheter ablation. Patients were divided into three groups based on the postoperative antiarrhythmic drug prescribed: dronedarone, amiodarone, or propafenone. A propensity score matching (PSM) analysis was performed as the primary analysis to compare early recurrence rates and drug-related adverse events. An inverse probability of treatment weighting (IPW) analysis was conducted as a sensitivity analysis.

**Results:**

Of the 408 patients, 65 (15.9%) experienced early recurrence. The early recurrence rate was significantly lower in the dronedarone group (9.2%) compared to propafenone (27.2%), but not significantly different from amiodarone (14.0%). The dronedarone group showed a lower recurrence rate of atrial flutter compared to both the propafenone and amiodarone groups (*p* < 0.05). After PSM, the recurrence rate of atrial flutter remained significantly lower in the dronedarone group compared to propafenone and amiodarone. Regarding drug-related adverse events, 73 patients (17.9%) experienced adverse reactions, with the amiodarone group showing a significantly higher incidence (24.2%) compared to the propafenone (13.6%) and dronedarone (11.8%) groups.

**Conclusion:**

In this retrospective study of patients with nonparoxysmal atrial fibrillation after catheter ablation, dronedarone and amiodarone showed a trend toward better efficacy than propafenone in preventing overall early recurrence. Dronedarone demonstrated a specific advantage in preventing early recurrence of atrial flutter.

## 1. Introduction

Atrial fibrillation (AF) is the most frequent sustained arrhythmia observed in clinical practice, characterized by disorganized atrial electrical activity and complete loss of atrial contractile function. The prevalence of AF in Chinese adults is currently about 1.6%, affecting nearly 20 million individuals [1]. Epidemiological studies indicate that the prevalence of AF significantly increases with age, particularly among individuals aged 65 and older. This condition not only substantially raises the risk of stroke, heart failure, and overall mortality but also profoundly impacts patients' quality of life [2]. Numerous studies have confirmed that catheter radiofrequency ablation can significantly improve the prognosis of patients with AF and has been recommended as a first-line treatment by both international and national guidelines [3, 4]. However, the recurrence rate of AF after catheter radiofrequency ablation remains relatively high. Previous studies reporting long-term outcomes have employed a broad range of blanking periods, typically between 72 h and 3 months postablation. During this period, patients are prone to atrial arrhythmias, such as AF, atrial flutter, and atrial tachycardia, some of which may spontaneously revert to sinus rhythm [5]. Arrhythmias occurring within the blanking period are referred to as early recurrence, while those occurring within the first year postprocedure are termed late recurrence, and recurrences beyond 1 year are classified as very late recurrence [6]. Statistics indicate that approximately 50% or more of patients experience recurrence within the blanking period, and 25%–40% of patients relapse within the first year after the procedure [7]. Although early recurrence does not necessarily indicate ablation failure, patients with early recurrence have a significantly higher failure rate compared to those without recurrence [8]. Early recurrence is also considered an independent risk factor for late recurrence [9]. Therefore, actively preventing early recurrence offers significant benefits to patients [10].

Antiarrhythmic drugs (AADs) are widely used following AF ablation. Research has shown that using these medications during the blanking period helps maintain sinus rhythm, improve atrial electrical remodeling, and reduce early recurrence [11, 12]. However, current guidelines have yet to specify the preferred drug for postoperative recurrence prevention. Propafenone and amiodarone are commonly used anti-AF medications, with amiodarone demonstrating significant efficacy in maintaining sinus rhythm and reducing postoperative recurrence [10]. Previous studies have indicated that using amiodarone during the blanking period is more effective in reducing early recurrence rates compared to propafenone [13]. Nevertheless, the clinical application of amiodarone is limited due to its long half-life and numerous adverse effects.

Dronedarone, a derivative of amiodarone, has been chemically modified to remove iodine atoms and add a methanesulfonamide group, thereby reducing amiodarone's thyroid, pulmonary, ocular, and neurological toxicities. Previous studies have shown that dronedarone significantly reduces the rate of first recurrence in AF patients [14], lowers the ventricular rate [15], and decreases hospitalization events due to cardiovascular causes [16]. Our study utilizes propensity score matching (PSM) analysis and inverse probability of treatment weighting (IPW) to compare the efficacy and safety of dronedarone, amiodarone, and propafenone in preventing early recurrence after AF catheter ablation, providing valuable insights for the selection of AADs.

## 2. Methods

The study was a single-center, retrospective, nonblinded study. The subjects were AF patients who underwent catheter ablation therapy for the first time at the Third Xiangya Hospital of Central South University from January 2021 to January 2023 (a total of 24 months). According to the inclusion and exclusion criteria below, a total of 408 patients met the study criteria. Preoperatively, transesophageal echocardiography (TOE) was performed to exclude left atrial thrombus, and pulmonary vein (PV) computed tomography (CT) was utilized to evaluate the anatomical structure of the left atrium. None of the patients in the cohort were receiving AADs prior to the procedure; medications such as beta-blockers were used for rate control only. As all patients were in nonparoxysmal AF at baseline, a specific preprocedural evaluation for organized atrial flutter was not applicable. Under local anesthesia, electrode catheters were inserted via the right internal jugular vein puncture into the coronary sinus, followed by the placement of a Swartz sheath via the right femoral vein puncture. After successful atrial septal puncture, heparinization was initiated with an initial intravenous dose of 70–100 IU/kg of heparin, with subsequent supplementation guided by the patient's activated clotting time (ACT) to maintain ACT within the range of 250–350 s.

High-density electrical mapping of the left atrium and bilateral PVs was performed using the PentaRay catheter (Johnson & Johnson), and pulmonary vein isolation (PVI) was achieved under the guidance of the CARTO3 system (Johnson & Johnson) using radiofrequency ablation with parameters of 50 W/43°C. For patients with nonparoxysmal AF, the HOT-AF strategy of the Third Xiangya Hospital was employed, which included bilateral PVI and structured ablation lines at the bottom and top of the left atrium. Following bilateral PVI, a modified BOX ablation technique was performed, which involved adding ablation lines at the top and bottom of the left atrium, with the bottom line connected to the isolation ring of the left PVs along the endocardial surface of the coronary sinus. Subsequently, the bidirectional block of the ablation lines was confirmed by electrophysiological examination, followed by targeted ablation of focal triggers. If sinus rhythm was not restored, electrical cardioversion was performed.

Our protocol also included a systematic approach to other potential arrhythmia sources. If a typical cavotricuspid isthmus (CTI)–dependent flutter was identified intraprocedurally, a CTI ablation was performed. Following the left atrial ablation, we routinely assessed for and ablated non-PV triggers, which included targeting focal sites in the superior vena cava, coronary sinus, and right atrium as clinically indicated.

After the operation, patients received at least 3 months of oral anticoagulant therapy, with the option to use warfarin or novel oral anticoagulants. The choice of AADs, such as propafenone, amiodarone, or dronedarone, was determined by the clinical physician based on the patient's underlying health condition. The recommended dose of propafenone is 150 mg per dose, three times a day; dronedarone is 400 mg per dose, twice a day; and amiodarone is 200 mg per dose, three times a day in the first week after surgery, twice a day in the second week, and once a day in the third week and thereafter.

### 2.1. Inclusion Criteria

1. Standard 12-lead electrocardiogram (ECG) or Holter monitor showing AF, meeting diagnostic criteria for nonparoxysmal AF.
2. Age ≥ 18 and ≤ 80 years.
3. Absence of left atrial thrombus on preoperative TOE.
4. Patients undergoing catheter ablation therapy and returning to sinus rhythm postoperatively.
5. Regular use of AADs for 3 months after ablation therapy and regular follow-up within 3 months postoperatively.

### 2.2. Exclusion Criteria

1. History of allergy to amiodarone, propafenone, or dronedarone.
2. New York Heart Association functional Class IV, left ventricular ejection fraction (LVEF) ≤ 40%, or recent acute decompensated heart failure requiring hospitalization.
3. History of previous radiofrequency ablation.
4. First-degree atrioventricular block with PR interval > 280 ms, second-degree or third-degree atrioventricular block, or sick sinus syndrome (unless a pacemaker is installed).
5. Bradycardia (average heart rate < 50 bpm within 24 h).
6. Concomitant use of potent CYP3A inhibitors.
7. QTc interval ≥ 500 ms, or concurrent use of drugs that prolong the QT interval and may increase the risk of torsades de pointes ventricular tachycardia.
8. Abnormal liver function: AST and ALT > 2.5 times the upper limit of normal; total bilirubin > 1.5 times the upper limit of normal.
9. History of amiodarone-related pulmonary toxicity.
10. Use of amiodarone or discontinuation of other AADs less than five half-lives within 4 weeks before ablation.
11. Congenital heart disease, acute myocardial infarction, unstable angina, and obstructive/hypertrophic obstructive cardiomyopathy.
12. Patients unable or unwilling to adhere to the prescribed AAD regimen for the full 3-month period, or those who were lost to follow-up.
13. Patients with incomplete clinical data.

### 2.3. Outcomes and Follow-Up

Inclusion in this study required completion of a standardized 3-month follow-up protocol. This protocol mandated monthly outpatient visits or telephone calls for the first 3 months postablation. Adherence to the prescribed AADs regimen was assessed via patient self-report during these monthly follow-ups.

During the follow-up period, a comprehensive examination was performed, including a complete blood count, liver and renal function tests, thyroid function tests, coagulation function tests, and a 12-lead ECG. In addition to the monthly ECGs, a mandatory 24-h Holter monitoring and an echocardiography were performed for all patients at the 3-month follow-up visit. Any recurrence of symptoms or adverse reactions was carefully recorded. Patients were instructed to undergo an immediate ECG if they experienced palpitations. For patients with documented recurrence or adverse reactions, adjustments to therapy were made at the discretion of the treating physician.

The primary outcomes include early recurrence of AF during the blanking period and adverse reactions caused by AADs. Early recurrence of AF is defined as sustained AF, atrial flutter, or atrial tachycardia lasting ≥ 30 s during the blanking period. Adverse reactions to AADs may include sinus bradycardia, prolonged QT interval, thyroid dysfunction, atrioventricular block, hepatic dysfunction, and renal dysfunction.

### 2.4. Statistical Analysis

PSM was implemented using R language and involved matching the three groups in a 1:1:1 ratio, with a caliper value set at 0.02, and estimating propensity scores using logistic regression. Based on clinical relevance and prior literature, a comprehensive set of baseline covariates potentially associated with both treatment selection and the outcome was included in the logistic regression model to calculate the propensity scores. After matching, the balance of covariates was assessed using *p* values and standardized mean differences (SMDs), with an SMD < 0.1 considered to indicate a negligible imbalance (Table S1). Data analysis is conducted using SPSS 24.0 and R to compare the data before and after matching. Normally distributed data are presented as mean ± standard deviation, nonnormally distributed data are presented as a median and interquartile range, and categorical data are presented as frequency and percentage. The significance of the three groups' data is analyzed using variance analysis, Kruskal–Wallis *H* test, and chi-square test for normally distributed, nonnormally distributed, and categorical data, respectively. Survival analysis is performed using the Kaplan–Meier method, and the cumulative maintenance rate of sinus rhythm among the three groups is compared using the log rank test.

Following PSM, univariate and multivariate logistic regression analyses are conducted to assess the impact of drug therapy on early recurrence postablation. To ensure the stability of the multivariate model, we checked for multicollinearity among the covariates using the variance inflation factor (VIF). A VIF value < 5 was considered indicative of no significant multicollinearity. A two-sided *p* value < 0.05 was considered statistically significant.

### 2.5. Sensitivity Analysis

To assess the robustness of our findings, we performed a sensitivity analysis with IPW. Stabilized weights were calculated for each patient based on a propensity score model identical to that used in the primary analysis. Covariate balance after weighting was confirmed by ensuring all absolute SMDs were below 0.1. The outcomes of early recurrence and its subtypes (AF and atrial flutter) were then reanalyzed in the weighted cohort using weighted logistic regression models.

## 3. Results

### 3.1. Baseline Characteristics

**Figure 1:**
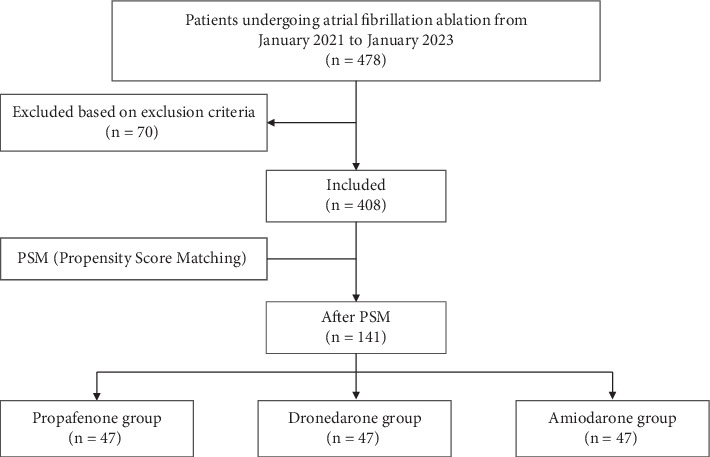
Flow diagram depicting patient disposition.

The study initially enrolled 478 patients. After excluding 25 lost to follow-up, 38 patients who had undergone radiofrequency ablation, three patients with failed ablation, and four patients with bradycardia, a total of 408 patients were ultimately included in the study. The inclusion and exclusion process is detailed in Figure 1, and the baseline characteristics are presented in Table 1. The average age of the three groups of patients was 60.25 ± 11.54 years, with 267 males (65.4%) and 141 females (34.6%). There were statistically significant differences (*p* < 0.05) among the three groups in terms of gender, average heart rate, brain natriuretic peptide (BNP) levels, left atrial diameter (LAD), right atrial diameter (RAD), right ventricular diameter (RVD), LVEF, history of heart failure, and history of thyroid disease.

### 3.2. Early Recurrence Between the Three Groups

Among 408 patients who underwent AF ablation, postoperative administration of propafenone was observed in 103 cases (25.2%), dronedarone in 119 cases (29.2%), and amiodarone in 186 cases (45.6%). Three-month follow-up revealed a total of 65 cases (15.9%) of early recurrence, with 28 cases (27.2%) in the propafenone group, 11 cases (9.2%) in the dronedarone group, and 26 cases (14.0%) in the amiodarone group, showing significant differences among the three groups (*p* < 0.001). The observed recurrent atrial arrhythmias included AF, atrial flutter, and atrial tachycardia.

**Figure 2:**
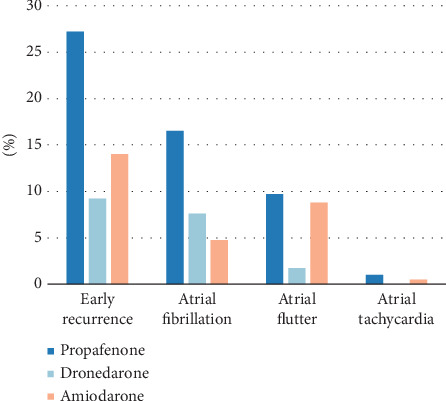
Bar chart comparing rates of early arrhythmia recurrence among the treatment groups before PSM.

In the comparison of early recurrence rates among the three patient groups, the postoperative early recurrence rate and incidence of AF in the dronedarone group and amiodarone group were significantly lower than those in the propafenone group (*p* < 0.05, Table 2 and Figure 2). Meanwhile, no significant differences were observed in the comparison of early recurrence and AF incidence between the dronedarone group and the amiodarone group. Additionally, the dronedarone group showed a significantly lower incidence of recurrent atrial flutter compared to the amiodarone group (*p* < 0.05). There were no statistically significant differences in the comparison of recurrent atrial tachycardia incidence among the three groups.

**Figure 3:**
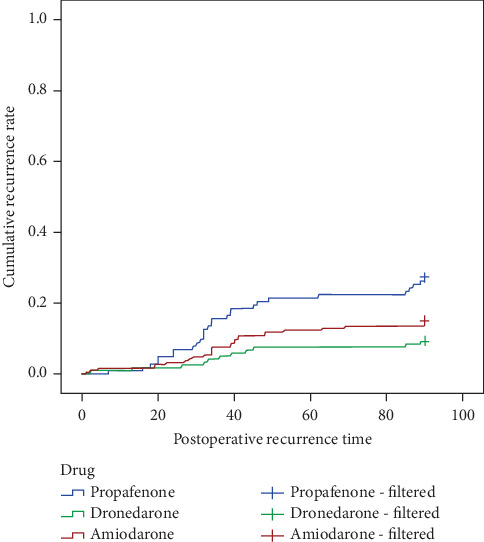
Kaplan–Meier curves for the cumulative incidence of early arrhythmia recurrence among treatment groups.

The average recurrence time across the three treatment groups was 39.92 ± 23.47 days. The Kaplan–Meier survival analysis was performed for further analysis (Figure 3). During the blanking period, the early recurrence rate in the propafenone group was significantly higher than that in the dronedarone group and the amiodarone group (*p* = 0.001, Table 3).

### 3.3. Adverse Drug Reaction Between the Three Groups

During the follow-up period, among the 408 patients, a total of 73 cases experienced adverse drug reactions related to AADs, including sinus bradycardia, QT interval prolongation, thyroid dysfunction, atrioventricular block, liver dysfunction, and renal dysfunction. For overall adverse reactions, there were 14 cases (13.6%) in the propafenone group, 14 cases (11.8%) in the dronedarone group, and 45 cases (24.2%) in the amiodarone group, with statistically significant differences among the three groups (*p* = 0.009). There were no significant differences in the incidence rates of sinus bradycardia, QT interval prolongation, atrioventricular block, liver dysfunction, and renal dysfunction among the groups. Additionally, thyroid dysfunction occurred in a total of 20 cases, with two cases (1.9%) in the propafenone group, zero cases in the dronedarone group, and 18 cases (9.7%) in the amiodarone group, showing statistically significant differences (*p* = 0.001, Table 4). When comparing the adverse drug reaction rates and incidence of thyroid dysfunction among the three drug groups, the amiodarone group showed significantly higher rates compared to the propafenone group (*p* = 0.032 and *p* = 0.013) and the dronedarone group (*p* = 0.007 and *p* = 0.001, Table 5). There were no significant differences between the propafenone group and the dronedarone group in these two indicators (*p* = 0.683 and *p* = 0.127, Table 5).

### 3.4. Early Recurrence and Adverse Drug Reaction After PSM

Given the significant baseline differences between the treatment groups (as shown in Table 1), PSM was performed to balance these covariates. A total of 141 patients (47 per group) were successfully matched. After matching, there were no longer any statistically significant differences in baseline characteristics among the three groups, indicating that a well-balanced cohort was achieved (all *p* > 0.05; Table 6).

**Figure 4:**
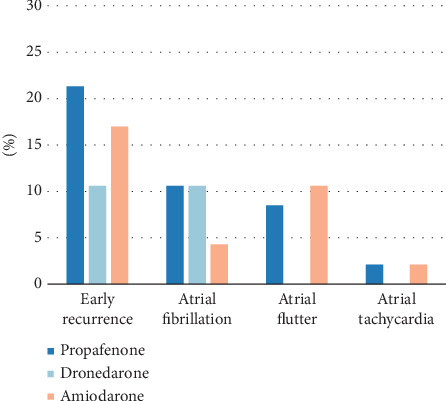
Bar chart comparing rates of early arrhythmia recurrence among the treatment groups after PSM.

After PSM, among a total of 141 patients, 23 cases (16.3%) experienced early recurrence. Specifically, there were 10 cases (21.3%) in the propafenone group, five cases (10.6%) in the dronedarone group, and eight cases (17.0%) in the amiodarone group. Regarding the recurrence of atrial arrhythmias, the recurrence rate of AF was 10.6% in both the propafenone and dronedarone groups, while it was 4.3% in the amiodarone group. There was no statistically significant difference in the early recurrence of atrial tachycardia among the three groups (*p* = 0.602, Table 7). For atrial flutter, the dronedarone group showed a lower early recurrence rate of atrial flutter compared to the propafenone group (*p* = 0.041) and the amiodarone group (*p* = 0.022, Table 7 and Figure 4).

### 3.5. Single-Factor and Multiple-Factor Logistic Regression Analysis

After conducting a single-factor logistic regression analysis of baseline data for three groups of patients matched by propensity score, it was found that LAD, RAD, LVEF, heart failure, and history of stroke, TIA, or thrombosis were significantly associated with early postoperative recurrence (Table 8). After conducting multiple-factor logistic regression analysis to adjust for relevant factors, the study revealed a significant association between postoperative use of AADs and early recurrence. Compared to dronedarone, patients who received propafenone postoperatively had a higher likelihood of early recurrence (*p* = 0.024). However, there was no statistically significant difference in early recurrence between postoperative use of amiodarone and dronedarone (Table 9).

Following PSM analysis, the adverse events were recorded across the three patient groups: four cases (8.5%) in the propafenone group, five cases (10.6%) in the dronedarone group, and eight cases (17.0%) in the amiodarone group. These differences did not reach statistical significance (*p* = 0.419). The incidence rates of sinus bradycardia, atrioventricular block, and hepatic dysfunction did not exhibit significant differences among the three groups, while the incidence rate of thyroid dysfunction in the amiodarone cohort (6.4%) was higher than in the propafenone group and the dronedarone group (Table 10).

### 3.6. Sensitivity Analysis

To assess the robustness of our primary findings, we conducted a sensitivity analysis using IPW on the entire cohort of 408 patients. After applying the weights, all baseline covariates were well balanced between the treatment groups (SMDs < 0.1; Table S2). The results of the weighted logistic regression analysis were consistent with the primary analysis from the PSM cohort. Compared to the dronedarone group, the propafenone group had a significantly higher odds of early recurrence (*p* = 0.004), while the amiodarone group showed no significant difference (*p* = 0.198). Similarly, for the outcome of atrial flutter, both propafenone (*p* = 0.029) and amiodarone (*p* = 0.015) were associated with a higher risk compared to dronedarone (Table S3). These findings confirm the robustness of our primary conclusions.

## 4. Discussion

In this study, we conducted a comparative analysis of the efficacy and safety of dronedarone, amiodarone, and propafenone for preventing early recurrence in patients with nonparoxysmal AF following catheter ablation. Our primary analysis, using PSM, revealed two key findings. First, while a clear trend favored dronedarone and amiodarone over propafenone for preventing any early recurrence, this difference did not reach statistical significance for the overall comparison after matching. Second, dronedarone was associated with a specifically and significantly lower rate of early atrial flutter recurrence compared to both other agents, a finding that remained robust after PSM and was consistent with our sensitivity analysis using IPW.

Previous studies have indicated that the early recurrence rate of AF following radiofrequency ablation can be as high as 50% [17], while late recurrence rates generally range between 20% and 30%, with nonparoxysmal AF exhibiting an even higher recurrence rate [18]. However, early recurrence does not necessarily signify treatment failure, as most early recurrences of arrhythmias may completely resolve after the blanking period [19]. The mechanisms underlying early and late recurrence of AF postablation differ. Research suggests that inflammatory responses related to the ablation procedure, such as myocardial edema, ischemia, necrosis, and local inflammation, may be key factors contributing to early recurrence by slowing atrial conduction [20]. Additionally, stable ablation scars typically form within 3 months postprocedure, implying that early postablation atrial arrhythmias may be related to immature ablation scars [21]. Furthermore, catheter ablation may also induce autonomic dysregulation, characterized by increased sympathetic and decreased parasympathetic activity, potentially leading to tachyarrhythmias [22]. Studies have shown that early recurrence is a reliable predictor of late recurrence after AF radiofrequency ablation, with 70% of patients experiencing late recurrence following early recurrence (Y. G. [23]). Therefore, preventing early recurrence is crucial for improving patient outcomes. Research indicates that amiodarone, propafenone, and dronedarone are effective in reducing early recurrence rates [24].

Our study suggests that in nonparoxysmal AF patients undergoing catheter ablation, dronedarone is associated with a lower early recurrence rate compared to propafenone, with no significant difference from amiodarone. The differences in efficacy may be attributed to (1) ion channel blockade: dronedarone and amiodarone exhibit broader inhibition of sodium, potassium, and calcium channels compared to propafenone, which primarily blocks sodium channels [25]; (2) autonomic modulation: dronedarone and amiodarone noncompetitively inhibit both *α*- and *β*-adrenergic receptors, whereas propafenone primarily acts as a competitive *β*-blocker [26]; and (3) anti-inflammatory properties: as phospholipase inhibitors, dronedarone and amiodarone possess notable anti-inflammatory effects, with amiodarone additionally protecting myocardial cells from oxidative stress [27].

Additionally, our study found that the early recurrence rate of atrial flutter was significantly lower in the dronedarone group than in both the propafenone and amiodarone groups. Given the extensive atrial ablation and transmural injury required for nonparoxysmal AF, the anti-inflammatory and autonomic modulation effects of dronedarone may contribute to their potential benefits in preventing early recurrence [28, 29]. The ATHENA trial further demonstrated that dronedarone reduced cardiovascular hospitalization and mortality risk compared to placebo, with post hoc analysis suggesting a reduced burden of nonparoxysmal AF and atrial flutter [30]. However, given the retrospective nature of our study, these findings should be interpreted with caution. Further prospective studies are needed to validate this specific role of dronedarone in postablation management.

In the historical context of AAD selection, propafenone has been widely recommended as a first-line treatment due to its lower incidence of adverse effects. In contrast, amiodarone, with its significant extracardiac toxicity, is primarily used in patients where propafenone is contraindicated [31]. Our study demonstrated that, compared to the dronedarone and propafenone groups, the amiodarone group had a higher rate of adverse drug reactions, particularly thyroid dysfunction, aligning with findings from Boriani et al. [32]. At the same time, our study did not observe major adverse events such as liver damage, pulmonary fibrosis, significant QT interval prolongation, or malignant arrhythmias associated with amiodarone or dronedarone. There were no significant differences in drug safety between dronedarone and propafenone [33]. After PSM, adverse event rates across all groups were similar, suggesting that baseline differences may have contributed to the observed disparities in the unadjusted analysis. Nonetheless, the risk of thyroid dysfunction remained uniquely elevated in the amiodarone group even after matching, highlighting its distinct side-effect profile. Additionally, while patients were extensively followed, data on some mild and less directly monitorable adverse effects, such as corneal microdeposits, photosensitivity, or fatigue, might remain incomplete.

### 4.1. Limitations

Several limitations of this study should be acknowledged. First, our primary analysis relied on PSM, which led to a significant reduction in sample size from 408 to 141 patients. This may have limited the statistical power of the primary analysis to detect more subtle differences and could affect the external validity of the findings. To address this, we performed a sensitivity analysis using IPW, which utilized the entire cohort. The consistency of the results between the PSM and IPW analyses strengthens the robustness of our conclusions. Nevertheless, the relatively small overall sample size limited subgroup analyses to explore treatment effects in different patient profiles.

Second, the retrospective, single-center design inherently carries risks of selection bias and residual confounding. While PSM aimed to mitigate these issues, some baseline imbalances persisted, and unmeasured factors such as medication adherence, lifestyle variables, or the specific rationale for physicians' choice of postoperative AADs based on prior drug history could still have influenced the outcomes.

Third, the absence of a no-drug control group prevents us from assessing the absolute efficacy of these AADs compared to no treatment. Our conclusions are therefore limited to the relative efficacy and safety among dronedarone, amiodarone, and propafenone.

Furthermore, the short 3-month follow-up period restricts our ability to evaluate long-term outcomes. Whether the observed reduction in early recurrence translates into a lower incidence of late recurrence remains an unanswered and clinically crucial question. The reliance on scheduled clinic visits and symptom-triggered ECGs, rather than continuous monitoring, may also have led to an underestimation of asymptomatic recurrences, introducing potential information bias.

Future research should ideally involve prospective, multicenter designs with larger sample sizes and extended follow-up periods to address these limitations. Incorporating continuous monitoring and conducting prespecified subgroup analyses would be crucial to validate our preliminary findings and explore treatment heterogeneity before they can inform clinical practice.

## 5. Conclusions

In this retrospective study of patients with nonparoxysmal AF after catheter ablation, dronedarone and amiodarone showed a favorable trend toward lower rates of overall early recurrence compared to propafenone. The key finding was that dronedarone was associated with a significantly lower incidence of early atrial flutter recurrence. These results suggest that dronedarone may be an effective option for the prevention of atrial tachyarrhythmias in the postablation blanking period.

## Figures and Tables

**Table 1:** Baseline characteristics of surgical patients.

**Characteristic**	**Propafenone (** **n** = 103**)**	**Dronedarone (** **n** = 119**)**	**Amiodarone (** **n** = 186**)**	**p**
Age (y)	63 (53–69)	62 (52.5–69)	63 (55–70)	0.813
Gender (female *n*, %)	46 (44.7)	35 (29.4)	60 (32.3)	0.039⁣^∗^
Hb (g/L)	131 (122–142)	130 (121–143.75)	131 (121–146)	0.802
Mean heart rate (bp/min)	74 (66–88.5)	79 (69–90)	83 (72–97)	0.006⁣^∗^
BMI (kg/m^2^)	24.3 (21.7–26.6)	25.05 (23.125–27.675)	25 (23.1–27.7)	0.058
BNP (pg/mL)	416 (106.61–1453.10)	523.54 (175.18–1735.77)	785.70 (290.3–1822.97)	0.003⁣^∗^
LAD (mm)	36.89 ± 5.84	37.28 ± 6.17	39.16 ± 5.89	0.012⁣^∗^
LVD (mm)	48 (44–49)	47 (45–50)	47 (44–51)	0.502
RAD (mm)	34 (31–38)	33 (31–38)	37 (32–40)	0.027⁣^∗^
RVD (mm)	30 (27–31)	30 (28–32)	31 (28–33)	0.030⁣^∗^
LVEF (%)	64 (59–69)	65 (60–69)	63 (50–68)	0.008⁣^∗^
Hypertension (*n*, %)	42 (40.8)	57 (47.9)	99 (53.2)	0.126
Diabetes (*n*, %)	19 (18.4)	19 (16)	50 (26.9)	0.052
Hyperlipidemia (*n*, %)	20 (19.4)	32 (26.9)	50 (26.9)	0.318
Coronary heart disease (*n*, %)	25 (24.3)	27 (22.7)	140 (24.7)	0.918
Heart failure (*n*, %)	34 (33)	38 (31.9)	83 (44.6)	0.041⁣^∗^
History of stroke, TIA, or thrombosis (*n*, %)	8 (7.8)	5 (4.2)	21 (11.3)	0.089
COPD (*n*, %)	1 (1)	3 (2.5)	4 (2.2)	0.686
Hypothyroidism (*n*, %)	2 (1.9)	16 (13.4)	1 (0.5)	0.001⁣^∗^
Hyperthyroidism (*n*, %)	10 (9.7)	8 (6.7)	2 (1.1)	0.003⁣^∗^
Renal insufficiency (*n*, %)	0 (0)	6 (5)	8 (4.3)	0.081
Peripheral vascular disease (*n*, %)	1 (1)	3 (2.5)	1 (0.5)	0.296
History of smoke (*n*, %)	23 (22.3)	23 (19.3)	48 (25.8)	0.415

*Note:* Hyperthyroidism and hypothyroidism are both past medical histories, and preoperative patients have thyroid function within the normal range.

⁣^∗^*p* < 0.05, indicating statistical significance.

**Table 2:** The early arrhythmia recurrence before PSM.

**Recurrence type**	**Propafenone (** **n** = 103**)**	**Dronedarone (** **n** = 119**)**	**Amiodarone (** **n** = 186**)**	**Overall ** **p** ** value**	**Prop vs. Dron**	**Prop vs. Amio**	**Dron vs. Amio**
Early recurrence	28 (27.2%)	11 (9.2%)	26 (14.0%)	< 0.001	< 0.001	0.006	0.217
Atrial fibrillation	17 (16.5%)	9 (7.6%)	9 (4.8%)	0.003	0.039	< 0.001	0.325
Atrial flutter	10 (9.7%)	2 (1.7%)	16 (8.6%)	0.028	0.008	0.753	0.012
Atrial tachycardia	1 (1.0%)	0 (0.0%)	1 (0.5%)	0.582	0.281	0.670	0.423

**Table 3:** Log-rank test.

	*χ* ^2^	**df**	**p**
Log rank (Mantel–Cox)	14.655	2	0.001⁣^∗^

⁣^∗^*p* < 0.05, indicating statistically significant differences.

**Table 4:** Adverse drug reactions.

	**Dronedarone (** **n** = 119**)**	**Propafenone (** **n** = 103**)**	**Amiodarone (** **n** = 186**)**	**p**
Sinus bradycardia (*n*, %)	4 (3.4)	7 (6.8)	17 (9.1)	0.150
QT interval prolongation (*n*, %)	1 (0.8)	0 (0.0)	4 (2.2)	0.264
Thyroid dysfunction (*n*, %)	0 (0.0)	2 (1.9)	18 (9.7)	0.001⁣^∗^
Atrioventricular block (*n*, %)	4 (3.4)	3 (2.9)	4 (2.2)	0.806
Liver dysfunction (*n*, %)	4 (3.4)	2 (1.9)	2 (1.1)	0.373
Renal dysfunction (*n*, %)	1 (0.8)	0 (0.0)	0 (0.0)	0.296
Overall adverse drug reactions (*n*, %)	14 (11.8)	14 (13.6)	45 (24.2)	0.009⁣^∗^

⁣^∗^*p* < 0.05, indicating statistically significant differences.

**Table 5:** The comparison of drug-related adverse reactions and thyroid dysfunction among the three groups.

	**Dronedarone (** **n** = 119**)**	**Propafenone (** **n** = 103**)**	**Amiodarone (** **n** = 186**)**	**p**
Thyroid dysfunction (*n*, %)	0 (0.0)	2 (1.9)	—	0.127
	—	2 (1.9)	18 (9.7)	0.013⁣^∗^
	0 (0.0)	—	18 (9.7)	0.001⁣^∗^

Drug-related adverse reactions (*n*, %)	14 (11.8)	14 (13.6)	—	0.683
	—	14 (13.6)	45 (24.2)	0.032⁣^∗^
	14 (11.8)	—	45 (24.2)	0.007⁣^∗^

⁣^∗^*p* < 0.05, indicating statistically significant differences.

**Table 6:** Baseline characteristics of patients after PSM.

**Characteristic**	**Dronedarone**	**Propafenone**	**Amiodarone**	**p**
Age (y)	64 (49–72)	63 (53–71)	63 (54–70)	0.958
Gender (female *n*, %)	14 (29.6)	14 (29.6)	21 (44.7)	0.216
Hb (g/L)	132.68 ± 16.05	132.64 ± 14.36	129.15 ± 21.66	0.539
Mean heart rate (bp/min)	76 (68–87)	74 (66–85)	82 (70–91)	0.355
BMI (kg/m^2^)	25.10 ± 3.82	24.38 ± 3.29	25.18 ± 2.88	0.443
BNP (pg/mL)	341 (117.18–1275.87)	384.2 (66.7–1905.38)	665.21 (290.3–1652.24)	0.302
LAD (mm)	35.83 ± 5.64	36.23 ± 5.25	37.19 ± 5.44	0.302
LVD (mm)	47 (45–50)	48 (45–50)	46 (42–49)	0.188
RAD (mm)	33 (31–38)	34 (31–38)	36 (31–40)	0.386
RVD (mm)	31 (28–32)	30 (27–32)	31 (27–32)	0.763
LVEF (%)	64 (59–69)	64 (59–69)	65 (57–70)	0.999
Hypertension (*n*, %)	21 (44.7)	21 (44.7)	20 (42.6)	0.972
Diabetes (*n*, %)	10 (21.3)	7 (14.9)	10 (21.3)	0.662
Hyperlipidemia (*n*, %)	14 (29.8)	13 (27.7)	14 (29.8)	0.966
Coronary heart disease (*n*, %)	9 (19.1)	14 (29.8)	11 (23.4)	0.479
Heart failure (*n*, %)	11 (23.4)	14 (29.8)	17 (36.2)	0.400
History of stroke, TIA, or thrombosis (*n*, %)	2 (4.3)	2 (4.3)	4 (8.5)	0.589
COPD (*n*, %)	2 (4.3)	1 (2.1)	1 (2.1)	0.773
Hypothyroidism (*n*, %)	0 (0)	2 (4.3)	1 (2.1)	0.360
Hyperthyroidism (*n*, %)	3 (6.4)	1 (2.1)	1 (2.1)	0.436
Renal insufficiency (*n*, %)	0 (0)	0 (0)	0 (0)	—
Peripheral vascular disease (*n*, %)	2 (4.3)	1 (2.1)	0 (0)	0.360
Smoking history (*n*, %)	12 (25.5)	13 (27.7)	5 (10.6)	0.090

**Table 7:** The early arrhythmia recurrence after PSM.

**Recurrence type**	**Propafenone (** **n** = 47**)**	**Dronedarone (** **n** = 47**)**	**Amiodarone (** **n** = 47**)**	**Overall ** **p** ** value**	**Prop vs. Dron**	**Prop vs. Amio**	**Dron vs. Amio**
Early recurrence	10 (21.3%)	5 (10.6%)	8 (17.0%)	0.373	0.159	0.601	0.370
Atrial fibrillation	5 (10.6%)	5 (10.6%)	2 (4.3%)	0.441	1.000	0.239	0.239
Atrial flutter	4 (8.5%)	0 (0.0%)	5 (10.6%)	0.083	0.041	0.726	0.022
Atrial tachycardia	1 (2.1%)	0 (0.0%)	1 (2.1%)	0.602	0.315	1.000	0.315

**Table 8:** Single-factor logistic regression analysis after PSM.

**Variables**	**β**	**SE**	**Wald's ** *χ* ^2^	**p**	**OR (95% CI)**
Age (y)	−0.021	0.019	1.268	0.260	0.979 (0.944, 1.016)
Male	0.072	0.535	0.018	0.892	1.075 (0.377, 3.065)
Female^a^					
Hb	0.024	0.015	2.402	0.121	1.024 (0.994, 1.055)
Mean heart rate	0.028	0.014	3.833	0.051	1.029 (1.000, 1.058)
BMI	0.075	0.075	1.583	0.208	1.099 (0.949, 1.272)
BNP	0.000	0.000	3.700	0.054	1.000 (1.000, 1.001)
LAD	0.180	0.056	10.472	0.001	1.197 (1.073, 1.335)
LVD	0.097	0.036	0.049	0.826	1.008 (0.940, 1.081)
RAD	0.154	0.050	9.490	0.002	1.167 (1.058, 1.287)
RVD	0.044	0.079	0.309	0.578	1.045 (0.895, 1.219)
LVEF	−0.081	0.024	11.363	0.001	0.922 (0.880, 0.967)
Hypertension	0.022	0.508	0.002	0.965	1.022 (0.378, 2.766)
Diabetes	0.217	0.612	0.126	0.723	1.242 (0.374, 4.125)
Hyperlipidemia	0.324	0.440	0.544	0.461	1.383 (0.584, 3.278)
Coronary heart disease	−0.392	0.503	0.607	0.436	0.676 (0.252, 1.811)
Heart failure	1.578	0.417	14.320	0.001	4.846 (2.140, 10.974)
History of stroke, TIA, or thrombosis	1.319	0.577	5.232	0.022	3.741 (1.208, 11.585)
COPD	0.059	1.172	0.003	0.960	1.061 (0.107, 10.544)
Hypothyroidism	0.474	1.241	0.146	0.703	1.606 (0.141, 18.280)
Hyperthyroidism	1.176	1.428	0.678	0.410	3.242 (0.197, 53.275)
Renal insufficiency	—	—	—	—	—
Peripheral vascular disease	—	—	—	—	—
Smoking history	0.243	0.472	0.265	0.607	1.275 (0.506, 3.214)
Dronedarone^a^					
Amiodarone	2.245	1.083	4.297	0.038	9.436 (1.321, 89.879)
Propafenone	2.388	1.077	4.921	0.027	10.895 (1.130, 78.783)

*Note:* The number of patients with renal insufficiency in all three drug groups was zero, and logistic regression analysis was not performed. The number of patients with peripheral vascular disease in the amiodarone group was zero, and logistic regression analysis was not performed.

^a^Control group.

**Table 9:** Multiple-factor logistic regression analysis after PSM.

**Variables**	**β**	**SE**	**Wald's ** *χ* ^2^	**p**	**OR (95% CI)**
Dronedarone^a^					
Propafenone	2.619	1.164	5.063	0.024	13.722 (1.402, 134.318)
Amiodarone	1.861	1.159	2.581	0.108	6.431 (0.664, 62.293)
LAD	0.132	0.084	2.472	0.116	1.141 (0.968, 1.346)
RAD	−0.054	0.076	0.496	0.481	0.948 (0.816, 1.100)
EF	−0.050	0.031	2.611	0.106	0.951 (0.896, 1.011)
Heart failure	0.025	0.712	0.001	0.972	1.026 (0.254, 4.137)
History of stroke, TIA, or thrombosis	0.603	0.930	0.421	0.516	1.828 (0.296, 11.311)

^a^Control group.

**Table 10:** Adverse drug reactions after PSM.

	**Propafenone (** **n** = 47**)**	**Dronedarone (** **n** = 47**)**	**Amiodarone (** **n** = 47**)**	**p**
Adverse drug reactions (*n*, %)	4 (8.5)	5 (10.6)	8 (17.0)	0.419
Sinus bradycardia (*n*, %)	2 (4.3)	3 (6.4)	3 (6.4)	0.876
Atrioventricular block (*n*, %)	2 (4.3)	1 (2.1)	1 (2.1)	0.773
Liver dysfunction (*n*, %)	0 (0.0)	1 (2.1)	1 (2.1)	0.602
Thyroid dysfunction (*n*, %)	0 (0.0)	0 (0.0)	3 (6.4)	0.047⁣^∗^

⁣^∗^*p* < 0.05, indicating statistically significant differences.

## Data Availability

The data that support the findings of this study are available from the corresponding author upon reasonable request.

## Nomenclature

AADs: antiarrhythmic drugs
ACT: activated clotting time
AF: atrial fibrillation
BMI: body mass index
BNP: brain natriuretic peptide
CI: confidence interval
CT: computed tomography
ECG: electrocardiogram
IPW: inverse probability of treatment weighting
LAD: left atrial diameter
LVEF: left ventricular ejection fraction
OR: odds ratio
PSM: propensity score matching
PVI: pulmonary vein isolation
RAD: right atrial diameter
RVD: right ventricular diameter
TIA: transient ischemic attack
TOE: transesophageal echocardiography

## Supporting Information

Supporting Information Additional supporting information can be found online in the Supporting Information section. Table S1: Standardized mean differences for baseline characteristics after propensity score matching (*n* = 141). Table S2: Standardized mean differences for baseline characteristics after inverse probability of treatment weighting (*n* = 408). Table S3: Sensitivity analysis of early recurrence using inverse probability of treatment weighting (*n* = 408).
