# Supplementary material for: Impact of Dronedarone on Early Recurrence After Catheter Ablation in Patients With Nonparoxysmal Atrial Fibrillation
Source: Cardiovasc Ther. 2025 Nov 14;2025:2585953. doi: 10.1155/cdr/2585953 (PMC12638134; doi:10.1155/cdr/2585953)
Supplement: Supporting Information — Additional supporting information can be found online in the Supporting Information section. Table S1: Standardized mean differences for baseline characteristics after propensity score matching (n = 141). Table S2: Standardized mean differences for baseline characteristics after inverse probability of treatment weighting (n = 408). Table S3: Sensitivity analysis of early recurrence using inverse probability of treatment weighting (n = 408). [file 2585953.f1.docx]

**Supplementary Material**

**Impact of Dronedarone on Early Recurrence After Catheter Ablation in Patients with Non-paroxysmal Atrial Fibrillation**

**Authors:** Xianglin Long^a^, Han Lv^a^, Jingliang Zhang^a^, Xiaoyan Wang^a^, Shan Tu^a^, Xu Deng^a^, Lixiong Zeng^a^, Wenzhi Luo^a^, Fei Ye^a^, Zhihui Zhang^a*^

a Department of Cardiology, The Third Xiangya Hospital of Central South University, Changsha, China.

*Corresponding address:

*Zhihui Zhang Ph.D. Department of Cardiology, The Third Xiangya Hospital of Central South University, No. 138, Tongzipo Road, Yuelu District, Changsha, China. E-mail: [zhangzhihui0869@csu.edu.cn](mailto:zhangzhihui0869@csu.edu.cn)

**Supplemental Table 1.** Standardized mean differences for baseline characteristics after propensity score matching (n = 141)

| **Characteristic** | **Dronedarone vs. Propafenone (SMD)** | **Dronedarone vs. Amiodarone (SMD)** |
| --- | --- | --- |
| **Demographics** |  |  |
| Age (y) | 0.04 | 0.06 |
| Gender (Female, %) | 0.00 | 0.09 |
| BMI (kg/m ²) | 0.08 | 0.03 |
| **Clinical Measurements** |  |  |
| Mean heart rate (bp/min) | 0.07 | 0.10 |
| Hb (g/L) | 0.01 | 0.05 |
| BNP (pg/mL) | 0.06 | 0.08 |
| **Echocardiographic Parameters** |  |  |
| LAD (mm) | 0.05 | 0.07 |
| LVD (mm) | 0.03 | 0.02 |
| RAD (mm) | 0.09 | 0.04 |
| RVD (mm) | 0.02 | 0.01 |
| EF (%) | 0.04 | 0.06 |
| **Comorbidities (%)** |  |  |
| Hypertension | 0.00 | 0.03 |
| Diabetes | 0.07 | 0.00 |
| Hyperlipidemia | 0.02 | 0.00 |
| Coronary heart disease | 0.08 | 0.05 |
| Heart failure | 0.05 | 0.09 |
| History of stroke/TIA/thrombosis | 0.00 | 0.06 |
| COPD | 0.03 | 0.03 |
| Hypothyroidism | 0.09 | 0.04 |
| Hyperthyroidism | 0.07 | 0.07 |
| Renal insufficiency | 0.00 | 0.00 |
| Peripheral vascular disease | 0.05 | 0.08 |
| **Lifestyle (%)** |  |  |
| Smoking history | 0.02 | 0.04 |

SMD = Standardized Mean Difference. A value of < 0.1 is generally considered to indicate a negligible difference between groups.

**Supplemental Table 2.** Standardized mean differences for baseline characteristics after inverse probability of treatment weighting (n=408)

| **Characteristic** | **Dronedarone vs. Propafenone (SMD)** | **Dronedarone vs. Amiodarone (SMD)** |
| --- | --- | --- |
| **Demographics** |  |  |
| Age (y) | 0.04 | 0.02 |
| Gender (Female, %) | 0.10 | 0.06 |
| BMI (kg/m ²) | 0.08 | 0.01 |
| **Clinical Measurements** |  |  |
| Mean heart rate (bp/min) | 0.08 | 0.09 |
| Hb (g/L) | 0.03 | 0.04 |
| BNP (pg/mL) | 0.09 | 0.07 |
| **Echocardiographic Parameters** |  |  |
| LAD (mm) | 0.07 | 0.09 |
| LVD (mm) | 0.02 | 0.04 |
| RAD (mm) | 0.08 | 0.10 |
| RVD (mm) | 0.05 | 0.07 |
| LVEF (%) | 0.06 | 0.10 |
| **Comorbidities (%)** |  |  |
| Hypertension | 0.07 | 0.05 |
| Diabetes | 0.02 | 0.09 |
| Hyperlipidemia | 0.07 | 0 |
| Coronary heart disease | 0.01 | 0.02 |
| Heart failure | 0.03 | 0.10 |
| History of stroke/TIA/thrombosis | 0.05 | 0.08 |
| COPD | 0.02 | 0.01 |
| Hypothyroidism | 0.09 | 0.05 |
| Hyperthyroidism | 0.04 | 0.09 |
| Renal insufficiency | 0.07 | 0.01 |
| Peripheral vascular disease | 0.03 | 0.04 |
| **Lifestyle (%)** |  |  |
| Smoking history | 0.04 | 0.07 |

SMD = Standardized Mean Difference. A value of < 0.1 is generally considered to indicate a negligible difference between groups.

**Supplemental Table 3.** Sensitivity analysis of early recurrence using inverse probability of treatment weighting (n=408)

| **Outcome** | **Comparison** | **Odds Ratio (OR)** | **95% Confidence Interval** | **P-value** |
| --- | --- | --- | --- | --- |
| Early recurrence | Propafenone vs. Dronedarone | 8.95 | [1.98, 40.52] | 0.004 |
|  | Amiodarone vs. Dronedarone | 1.76 | [0.75, 4.14] | 0.198 |
|  |  |  |  |  |
| Atrial fibrillation | Propafenone vs. Dronedarone | 1.28 | [0.51, 3.22] | 0.597 |
|  | Amiodarone vs. Dronedarone | 0.59 | [0.19, 1.80] | 0.360 |
|  |  |  |  |  |
| Atrial flutter | Propafenone vs. Dronedarone | 3.80 | [1.15, 12.56] | 0.029 |
|  | Amiodarone vs. Dronedarone | 3.55 | [1.28, 9.84] | 0.015 |

(Dronedarone as the reference group)
